# A Synergetic R-Shiny Portal for Modeling and Tracking of COVID-19 Data

**DOI:** 10.3389/fpubh.2020.623624

**Published:** 2021-01-27

**Authors:** Mahdi Salehi, Mohammad Arashi, Andriette Bekker, Johan Ferreira, Ding-Geng Chen, Foad Esmaeili, Motala Frances

**Affiliations:** ^1^Department of Mathematics and Statistics, University of Neyshabur, Neyshabur, Iran; ^2^Department of Statistics, Ferdowsi University of Mashhad, Mashhad, Iran; ^3^Department of Statistics, University of Pretoria, Hatfield, South Africa

**Keywords:** COVID-19, dashboard, gompertz growth model, logistic growth model, moran's index, open science, r, shiny

## Abstract

The purpose of this paper is to introduce a useful online interactive dashboard (https://mahdisalehi.shinyapps.io/Covid19Dashboard/) that visualize and follow confirmed cases of COVID-19 in real-time. The dashboard was made publicly available on 6 April 2020 to illustrate the counts of confirmed cases, deaths, and recoveries of COVID-19 at the level of country or continent. This dashboard is intended as a user-friendly dashboard for researchers as well as the general public to track the COVID-19 pandemic, and is generated from trusted data sources and built in open-source R software (Shiny in particular); ensuring a high sense of transparency and reproducibility. The R Shiny framework serves as a platform for visualization and analysis of the data, as well as an advance to capitalize on existing data curation to support and enable open science. Coded analysis here includes logistic and Gompertz growth models, as two mathematical tools for predicting the future of the COVID-19 pandemic, as well as the Moran's index metric, which gives a spatial perspective via heat maps that may assist in the identification of latent responses and behavioral patterns. This analysis provides real-time statistical application aiming to make sense to academic- and public consumers of the large amount of data that is being accumulated due to the COVID-19 pandemic.

## Introduction

COVID-19 needs no introduction in this day and age, as this disease has caused pandemic havoc around the globe since early January 2020. Even in so-called “first world countries,” the virus has caused a drastic focus on public health planning as well as emergency response measures (1). Without data—of the countries themselves, but also data of similar countries for comparison's sake—this drastic focus and emergency measures might be in vain if the overarching global effect is not constantly monitored (2, 3).

Rhodes et al. (4) argues that emergencies in the public health realm necessitates projections to produce evidence in response to such novel viral outbreaks (including, but not limited to an outbreak such as COVID-19). As part of the response to this ongoing global public health uncertainty, an interactive web-based dashboard has been developed for any end-user to be able to visualize and engage with the massive amount of data being accumulated in this unprecedented time. Such interfaces has been developed previously for other diseases as well as in climate change contexts, see (5–7). It is of utmost importance to support open science during the era where not only academics, but also the public, is bombarded by data on many fronts. We selected the R software package since it has a massive set of packages for visualization, importing and manipulating data; and forms the building blocks that we need to create this interactive dashboard with special characteristics to visualize and analyse the COVID-19 data. Shiny is an R package that makes it easy to build interactive web applications (apps) directly from R-based statistics computation and graphics. The number of users of R, as a statistical computational software, is growing up rapidly due to its wide variety of benefits such as being free, open source, and available on every major platform. The Shiny apps deployed are managed by “shinyapps.io”. It hosts each app on its own virtualized server (called an instance). The bundle size that can be uploaded is limited to 1 GB for the free accounts, while our dashboard, called “COVISA-19” (**COV**ID **I**ran **S**outh **A**frica-19), enjoys the “3X-Large” instance which provides an 8GB-memory for it, as a result, it can be loaded relatively fast (8).

This app provides a dashboard based on COVID-19 data as collected by the World Health Organization (WHO) and the Center for Disease Control (CDC) in the US. It has similar features as other such dashboards: to visualize the massive amounts of data that is mostly being recorded in real time (at least daily) (2). However, the particular power of this dashboard is 3-fold: producing downloadable plots of COVID-19 counts (infected, recovered, deceased) which user can sort by country/continent; an interactive setting, where users can shift days since first infection etc. on a scale and see how the data changed over time; and finally forecasting COVID-19 related cases using the logistic growth model (LGM) as well as the Gompertz growth model (GGM). In this way, a focus on a visual- as well as interactive level is available for the end user. Interactive tools make this dashboard a valuable addition to the growing body of knowledge relating to COVID; not only on an academic level, but also on a broader public engagement level. Moreover, the dashboard reflects both absolute counts and relative counts. The former represents the actual counted number of cases (confirmed, deaths, recoveries) for each region/country while the latter places the absolute counts in context with regards to the population size of that region (per 1 million residents) to consider the population density feature as well.

This dashboard has the potential to support policy making and decisions by having the option of user specified visual comparisons between countries for bilateral agreements (the BRICS countries—Brazil, Russia, India, China, and South Africa—and ensuing trade partnerships, for example), inter-country support to back up data/visualize current reports in convenient web based environment, and to support public education about the usage of data and the visualization thereof. Isheloke (9) describes the advent of the fourth industrial revolution and the intertwining nature of COVID-19 with the digital economy. The insight that the tools and functionalities in this dashboard bring for BRICS countries, for example, is tantamount is how policy and government guides deployment of handling the environment around us, and how government entities can put sound estimates on future planning based on observation and success of responding to numbers that emanate from the COVID-19 pandemic. In fact, Solberg & Akufo-Addo discusses how the pandemic has given rise to renewed investment into intellectual capital of the Sustainable Development Goals; this paper aims to assist in addressing this crucial gap of continued public knowledge and understanding of possible trends within this global public health crisis (10). In section Method we describe the design and the development of this paper that support this dashboard; Section Results we illustrate some special characteristics, and Section 4 contains some discussion and conclusions.

## Methods

**Figure 1:**
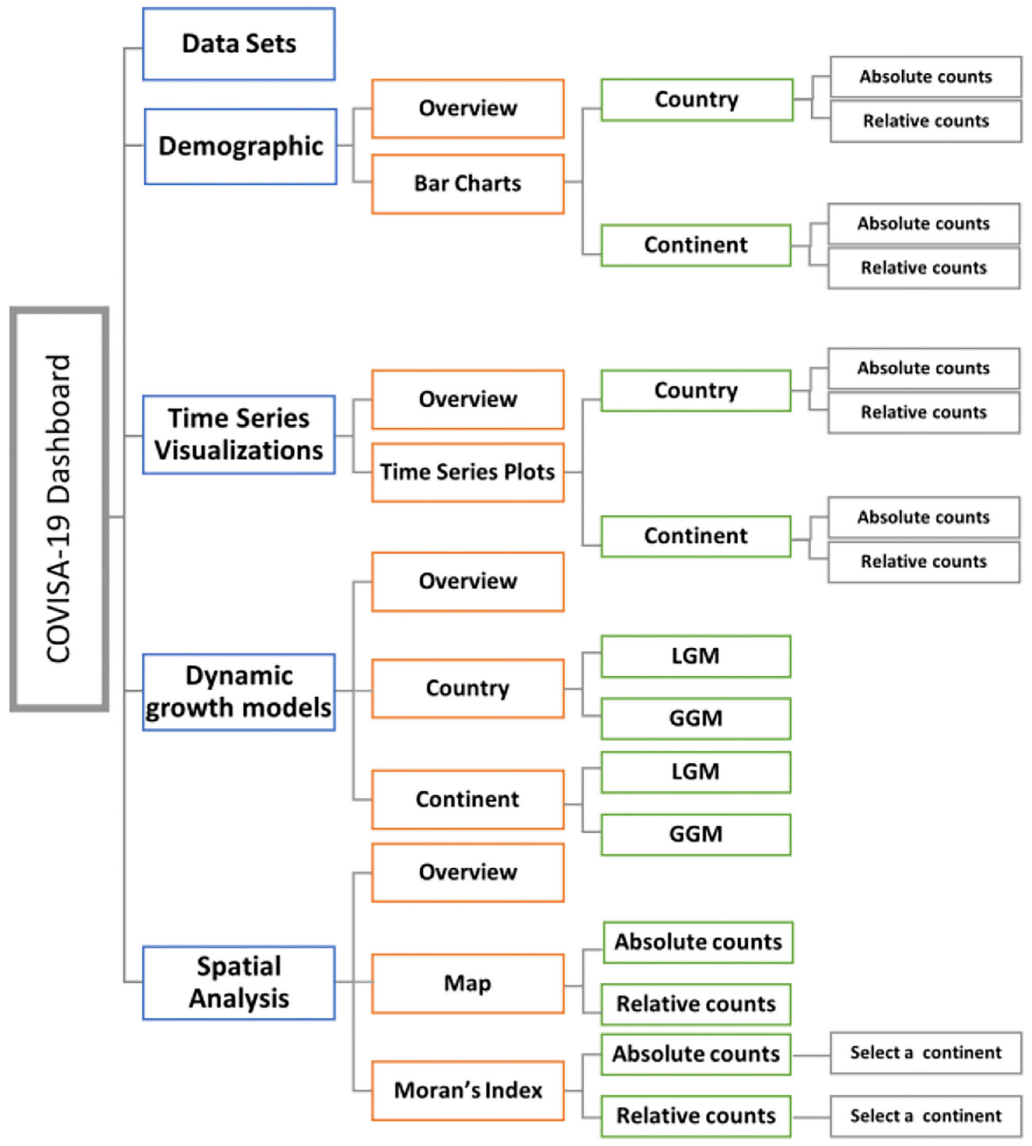
Workflow and modules of interactive web-based dashboard for COVID-19 data.

**Figure 2:**
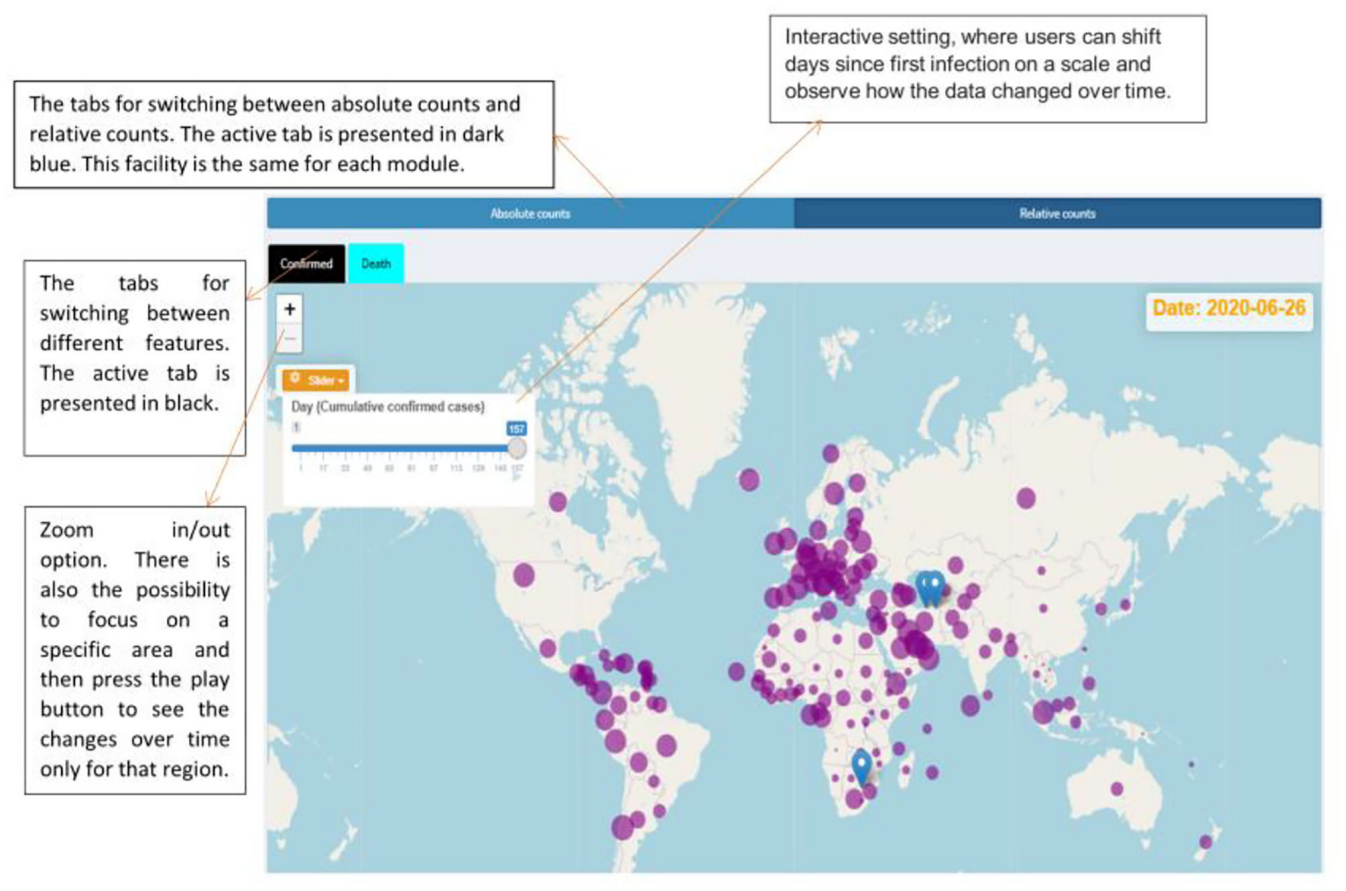
Facilities provided by the dashboard and the spread of the COVID-19 over the world up to 26 June 2020 based on the relative counts.

The dashboard is designed as a cross-platform web-browser accessible dashboard (portal) to display massive data in five dimensions (modules) of interest for the end user: data sets; demographic; time series plots; growth modeling; and spatial analysis. The R Shiny environment provides a platform for end-users to interact and visualize the data according to their needs. The workflow of the modules of this interactive web based dashboard is illustrated in Figure 1, followed by descriptions in Figure 2.

### Module 1: Data Set

This module provides the whole raw data and makes it possible to search a country of interest's name for obtaining the associated data by date for that specific country. The data are obtained from the online repository GitHub (https://pomber.github.io/covid19/timeseries.json) which transforms the data from Johns Hopkins University Center for Systems Science and Engineering (CSSEGISandData/COVID-19) into a json file. It is captured per country per day, which results in a large data frame. This data source includes information since January 22, 2020 up to the past 24 h (excluding the current day). It is automatically updated three times a day using GitHub Actions. Data calibration is not required in this instance as a shared GitHub repository is used for this visual purpose.

### Module 2: Demographic

This module presents bar charts that can indicate how different regions compare in terms of reported numbers of positive diagnoses (confirmed), deaths, and recovery counts. Here, one can choose between country/continent level and then absolute counts and relative counts.

### Module 3: Time Series Visualizations

The time series plots have been provided in this module per country per continent. There is a possibility here to compare the trend plots of different countries. Here also, one can choose between absolute counts and relative counts.

### Module 4: Dynamic Growth Models

In this menu, we have provided two dynamic models for forecasting the future of the pandemic. To this end, the LGM as well as the GGM, as two special cases of the generalized logistic curve, are fitted on the absolute cumulative counts of the confirmed cases.

### Module 5: Spatial Analysis

We present a spatial map indicating counts of confirmed cases, death cases, and recoveries. One can choose between absolute counts and relative counts. Furthermore, global Moran's index is also added for comparison between continents.

## Results

**Figure 3:**
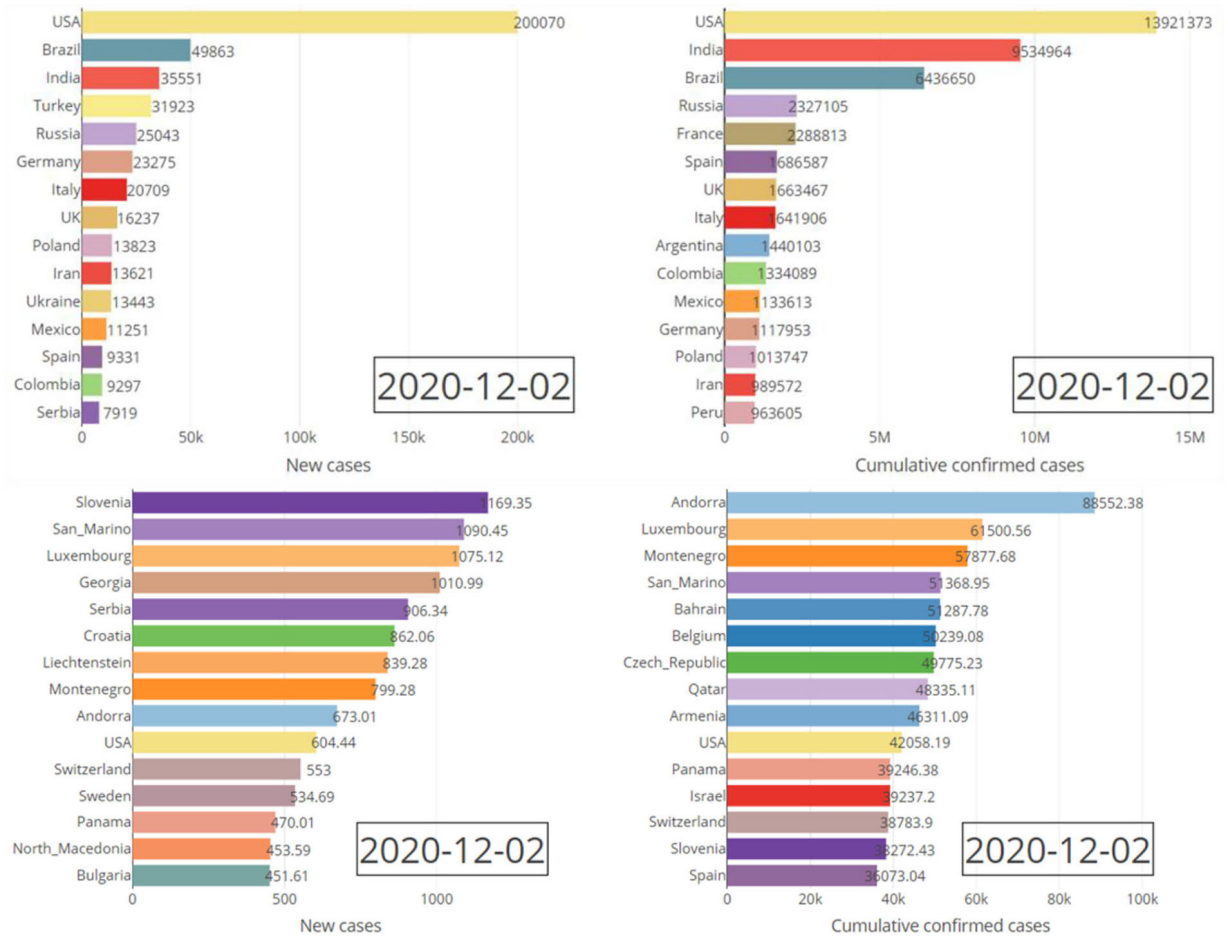
Absolute (top pane) and relative (bottom pane) daily/cumulative confirmed cases for the top fifteen countries on 2 December 2020.

**Figure 4:**
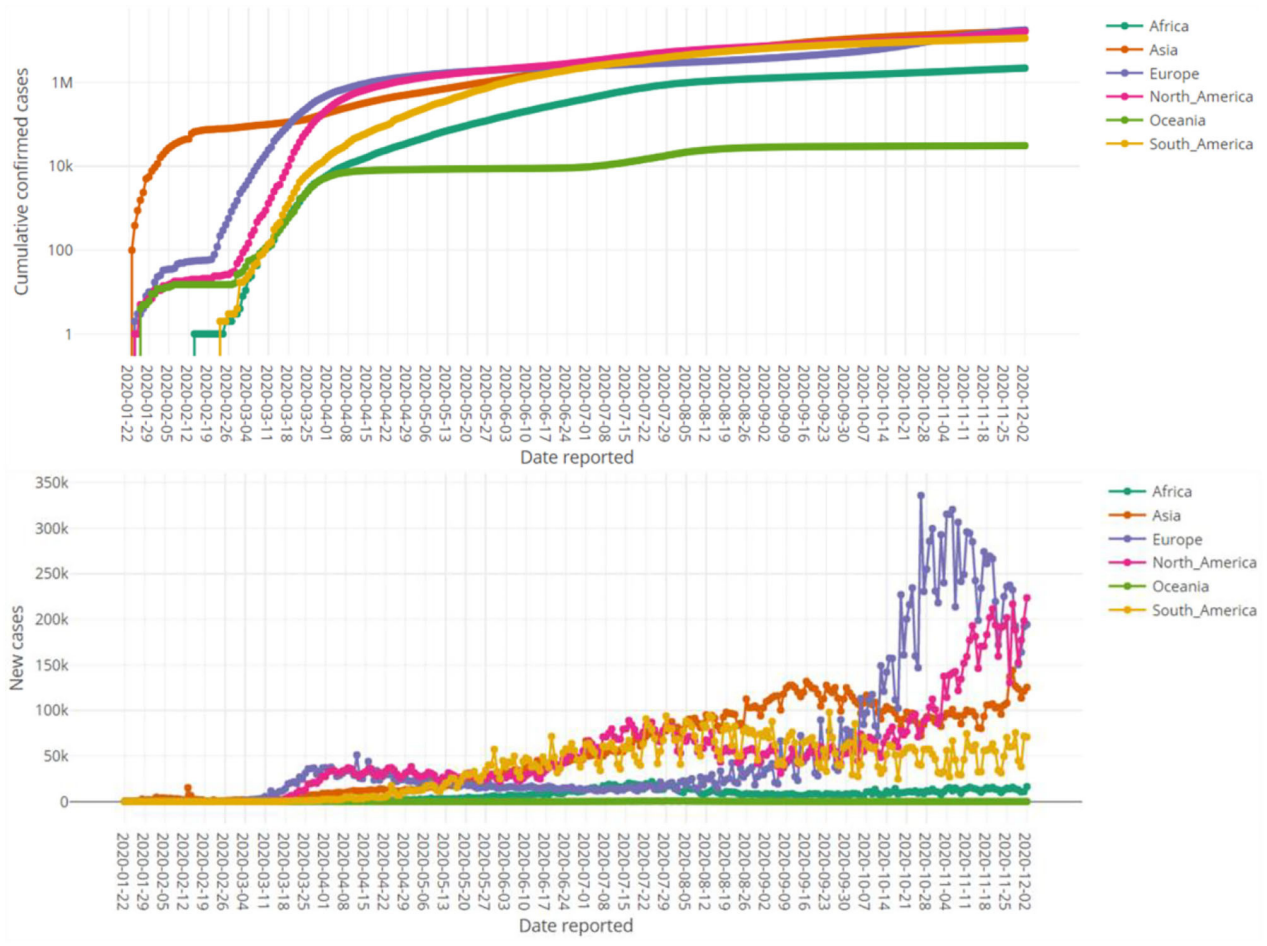
Time series plots of absolute cumulative confirmed cases in logarithmic scale (top pane) and daily confirmed cases (bottom pane) for various continents up to 2 December 2020.

**Figure 5:**
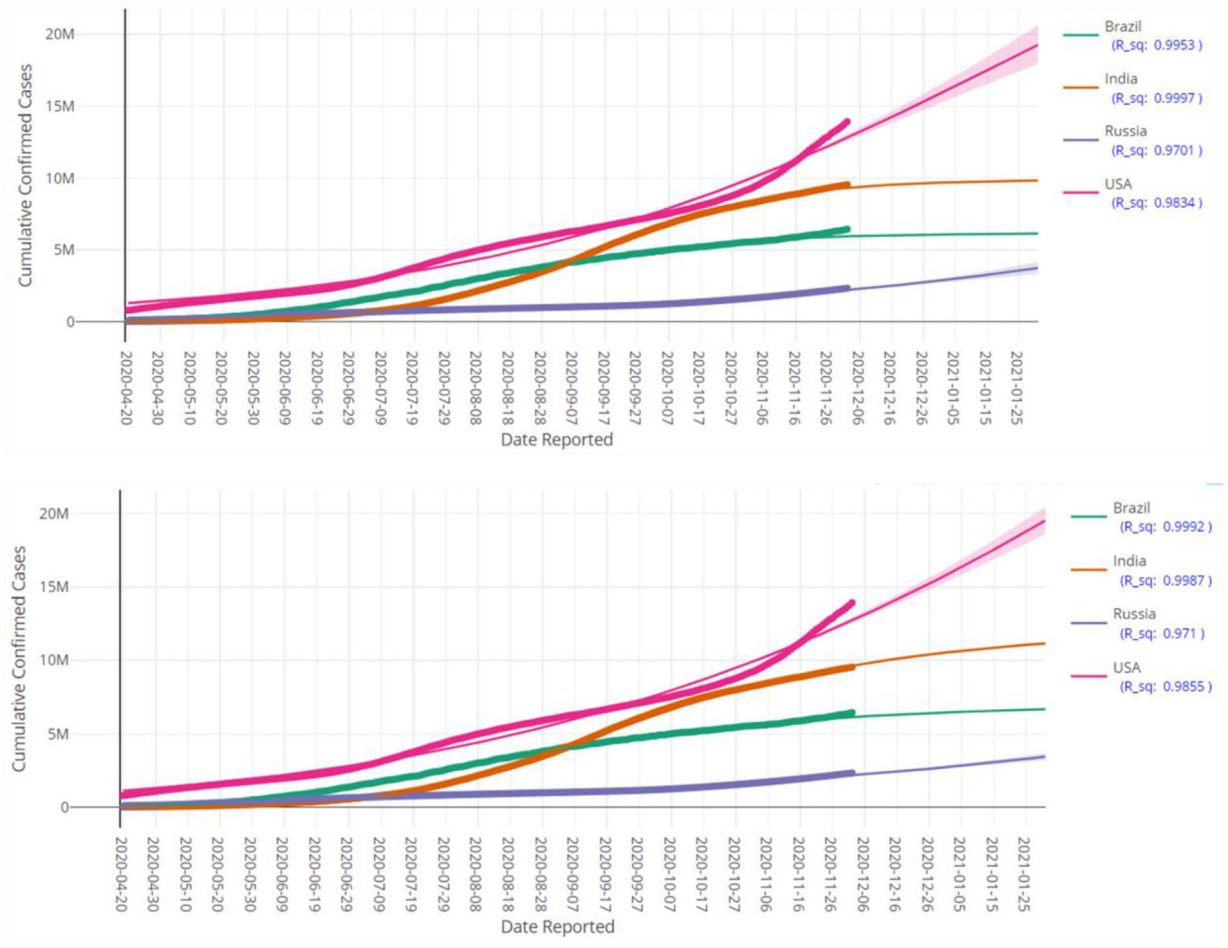
Prediction of cumulative confirmed cases in various countries up to 28 January 2021 based on LGM (top pane) and GGM (bottom pane). Each fitted curve is surrounded by a shadow area representing a 95% confidence interval. Each fitted curve is surrounded by a shadow area representing a 95% confidence interval.

**Figure 6:**
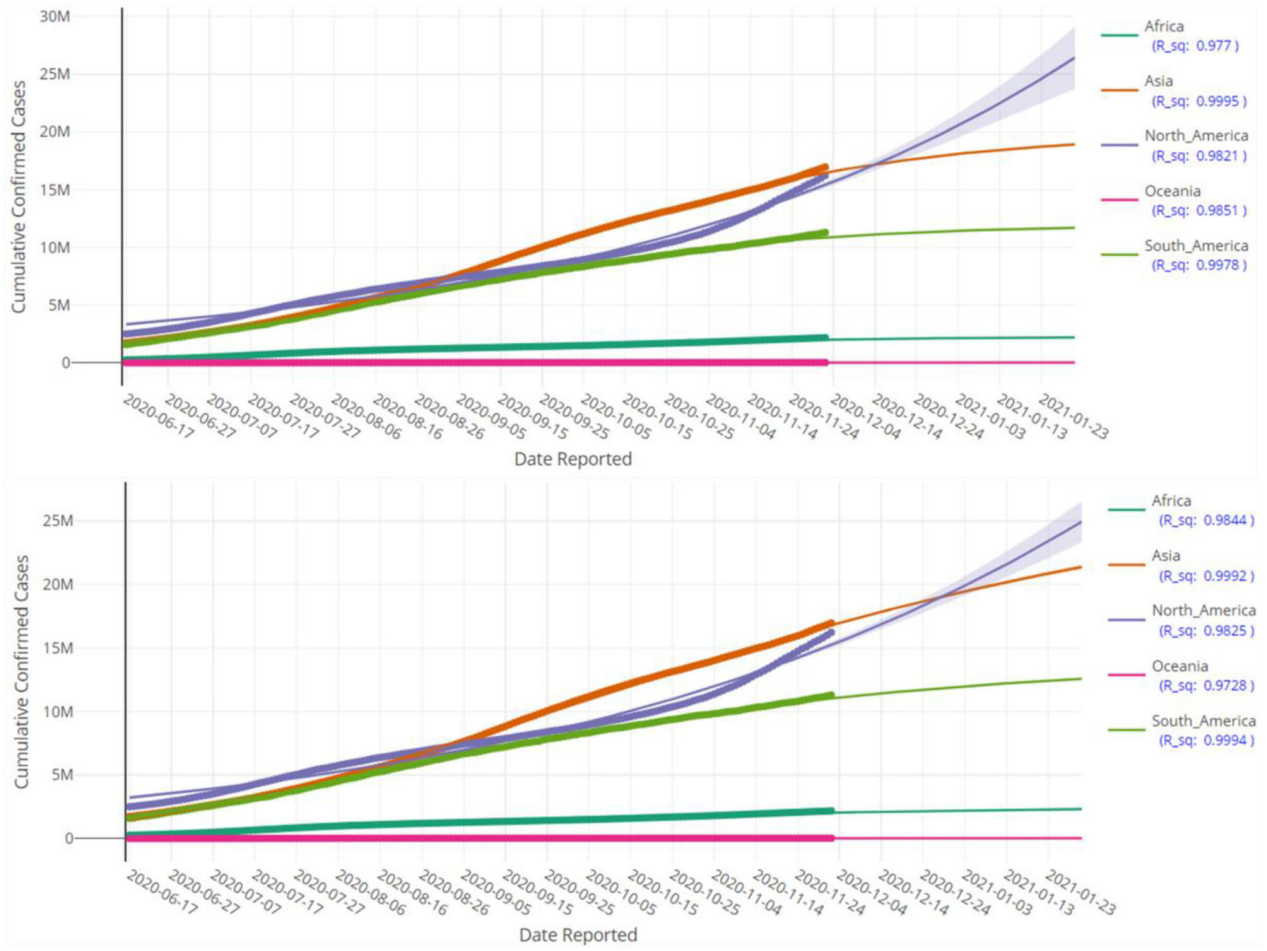
Prediction of cumulative confirmed cases in various continents up to 28 January 2021 based on LGM (top pane) and GGM (bottom pane). Each fitted curve is surrounded by a shadow area representing a 95% confidence interval.

**Figure 7:**
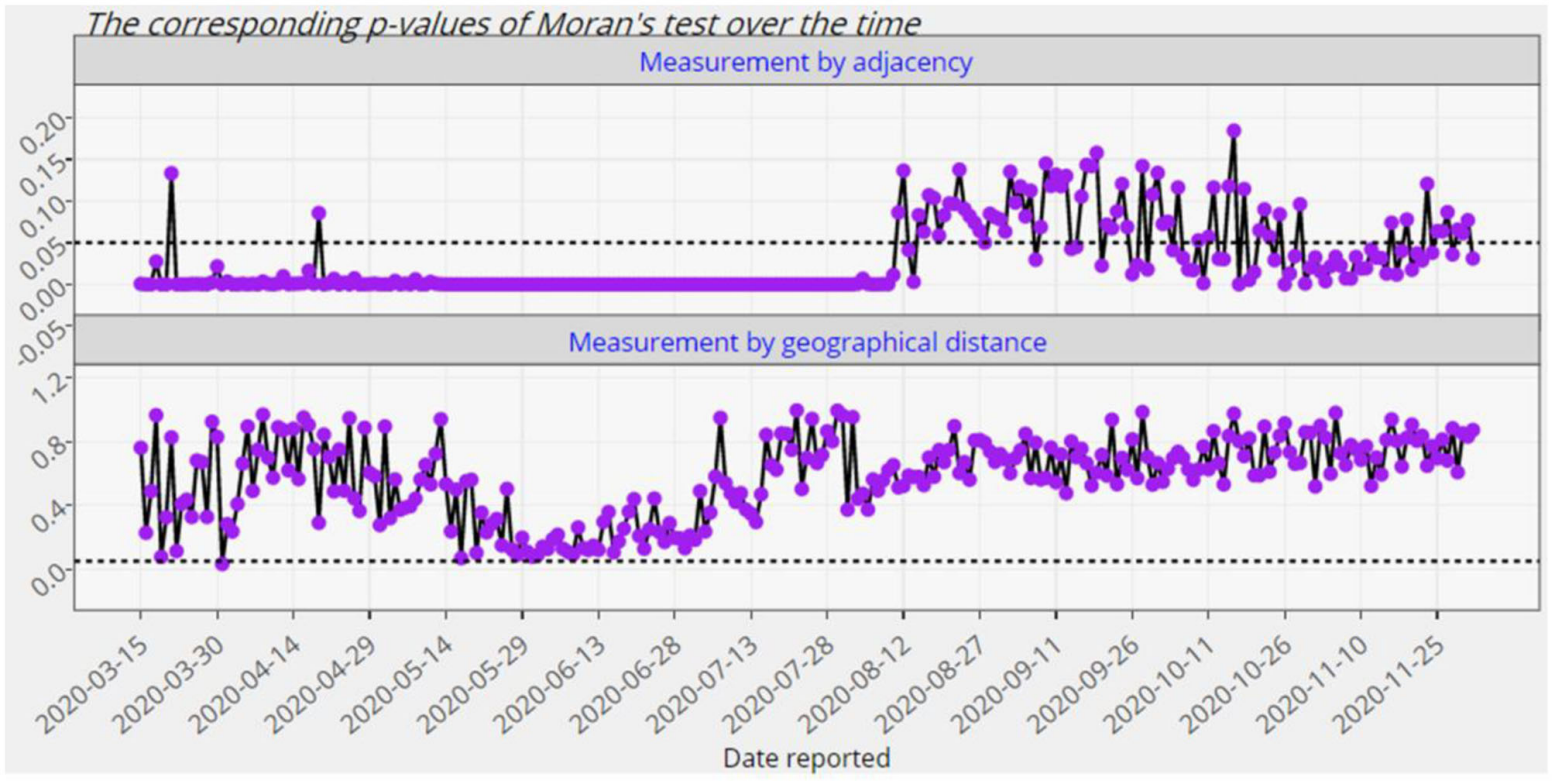
The p-values of the global Moran significance test for Europe from 15 March 2020 up to 2 December 2020.

**Figure 8:**
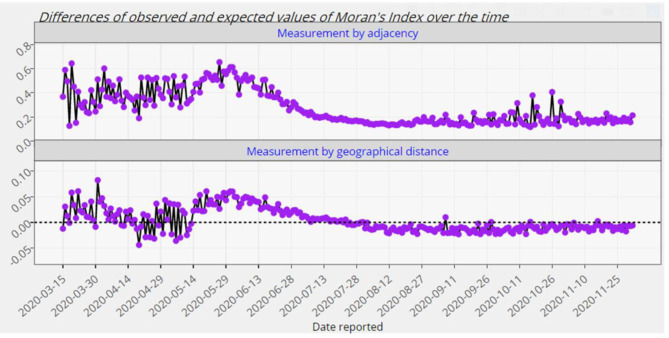
The differences of the observed and expected global Moran's index for Europe from 15 March 2020 up to 2 December 2020.

In this section the characteristics of this dashboard is demonstrated through various figures. All of the figures obtained here are directly downloadable from our R Shiny portal by any end-user. Various producible plots for both absolute and relative counts in the “Bar Charts” sub-module are displayed by Figure 3. Some producible plots for both absolute and relative counts in the “Time Series Plots” sub-module are displayed in Figure 4. Figures 5, 6 illustrate fitted growth models and the corresponding predictions for some countries and continents. Figures 7, 8 gives the associated results of Moran's spatial correlation test for Europe continent as an illustrative example.

### Visualization

Figure 3 compares the top fifteen affected countries by the COVID-19 based on both the absolute counts and relative counts. It reveals the stark difference between absolute and relative counts considering number of cases per 1 million residents of listed countries. Figure 4 shows the trend of the counts of the confirmed cases over the time for continents.

### Modeling Aspects

In this section, two often considered growth models that are incorporated in the dashboard, are briefly described, as well as the Moran's index.

#### Growth Models

The generalized logistic growth model and its variants are frequently used for dynamic modeling in many areas such as physics, chemistry, forestry, etc. It is also used in the medicine; particularly for examining disease spread, modeling of growth of tumors and modeling bacterial cells within a population. Now suppose that **N(t)** is the absolute cumulative counts of the confirmed cases of a given region. For our purpose, we follow the Richards' differential equation given by Richard's (11).

(1) $$\begin{array}{r} \frac{dN(t)}{dt}=\frac{1}{k}(1-{\left(\frac{N(t)}{\alpha }\right)}^{\nu })\;N(t) \end{array}$$

where **N**(**t**_**o**_)**=N**_**o**_, **α** denotes the maximum capacity or total population in the current context, **α, ν>**0, to obtain the generalized logistic growth model

(2) $$\begin{array}{r} N(t)=\frac{\alpha }{{\left(1+Ce^{-\frac{\nu t}{k}}\right)}^{\frac{1}{\nu }}}\;(1) \end{array}$$

with $\text{C}={\left(\frac{\alpha }{{\text{N}}_{0}}\right)}^{\nu }-1$. As mentioned earlier, Module 4 deals with fitting two dynamic models LGM and GGM for forecasting the future of the COVID-19 pandemic. Indeed, they are both special cases of the generalized growth model given by (1). More specifically, substituting ν = 1 in (1) yields the former, while the latter is a limiting case and is obtained from (1) as ν → 0^+^. In fact, although both of the models are sigmoid-shaped and bounded between two asymptotes, but have some different features as well. For instance, the inflection point in the LGM is exactly located at the mid of the asymptotes and there is a radial symmetry with respect to this point, while this properties does not hold for GGM. Thus, it is recommended that both of them should be fitted on the actual data (12).

Indeed, fitting the two well-known Richards' growth models, namely, the LGM and GGM, to the actual data is conducted by non-linear coding procedures in the R software. The question arises of how to make sure whether the forecasts are close to the real unobserved data. We point to deal with the differences and come to a significant amount of accuracy in forecasting. We estimated the models' parameters by minimizing the non-linear least-squares error. This way, the models are calculated using a non-parametric method. The underlying distribution does not affect estimation because the environmental assumptions may not ideal for fitting Richards' growth models.

Among the existing papers employing the generalized growth model and its variants on the COVID-19 data, some use the logistic growth equation to describe the process on a macroscopic level (13), others review the epidemic virus growth and decline curves in China using the phenomenological logistic growth model (14), and others who applied Moran's index for testing the significance of spatial auto-correlation and LGM for prediction of COVID-19 spread for the provinces of South Africa after lockdown (15). Also, Yu et al. (16) investigated the use of Gumbel growth modeling in forecasting in contrast to the logistic growth equation.

Figures 5, 6 display the fitted growth models LGM and GGM (the solid lines) on the observed values of cumulative confirmed cases (the points) as well as prediction of the future of the COVID-19 pandemic for some selected regions. The values of *R*^2^ for each model is close to the one, as a result, the adequacy of the models are confirmed. As mentioned before, the LGM and GGM can complement each other and both should be fitted on the data. Figures 5, 6 also support this fact and the evidence suggests that both of them are fitted well (but with different predictions) for the considered regions.

#### Spatial Autocorrelation

A spatial association or spatial autocorrelation measures how distance influences a particular variable. In other words, it quantifies the degree of which objects are similar to nearby objects. Variables are said to have a positive spatial autocorrelation when similar values tend to be nearer together than dissimilar values, otherwise, they are said to have a negative spatial autocorrelation when dissimilar values tend to be nearer together than similar values. The Moran's index, originally defined by Moran (17), is a measure of spatial autocorrelation which can be used to find spatial hotspots or clusters. It has been defined as the measure of choice for scientists, specifically in environmental sciences, ecology, and public health [see Zhang et al. (18)]. Moran's Index has a local and global representation: the global Moran's index is a global measure for spatial autocorrelation while the local Moran's index examines the individual locations, enabling hotspots to be identified based on comparisons to the neighboring samples. The global Moran's index *I* takes a value on in the interval [−1, 1] and *I* = 0 shows no spatial correlation between the sub-regions for the underlying feature. The values of global Moran's *I* near +1 indicate a sort of clustering, while the values close to −1 indicate outliers exist.

In Module 5 of COVISA19, we calculate the global Moran's index for countries of a given continent based on daily confirmed cases variable. More specifically, suppose that we have *d* countries in a given continent and the pair (*N*_*i*_, *N*_*j*_) stands for the total confirmed cases of two countries, *i, j* = 1, ⋯ , *d*. Assume further that the spatial weight *w*_*ij*_ quantifies the level of closeness between the countries *i* and *j*. Then, the global Moran's index is defined by

(3) $$\begin{array}{r} I=\frac{d}{S_{o}}.\frac{\sum_{i=1}^{d}\sum_{j=1}^{d}w_{ij}\left(N_{i}-\overset{-}{N}\right)\left(N_{j}-\overset{-}{N}\right)}{\sum_{i=1}^{d}{\left(N_{i}-\overset{-}{N}\right)}^{2}},\;i\neq j \end{array}$$

where $\overset{-}{N}=n^{-1}\sum_{i=1}^{n}N_{i}$ is the average of the counts of confirmed cases in that continent and $S_{o}=\sum_{i=1}^{n}\sum_{j=1}^{n}w_{ij}$. There are a couple of methods to identify the weights. Here, we employ the 0–1 adjacency as well as the geographical distance weight matrices [see e.g., (19) for more details]. Besides the values of the global Moran's *I* given by (2), Module 5 provides the corresponding p-values to evaluate the statistical significance of the spatial autocorrelation over time. The null hypothesis of this test states that there is no spatial clustering (or dispersion) of COVID-19 confirmed cases associated with the countries of a given continent. It is to be noted that the above computations are carried out based on both absolute counts and relative counts. Figures 7, 8 exhibit only an instance from this module.

As it is observed from Figures 7, 8, the European countries with touching borders have affected positively on each other from the mid of March up to the first week of August. Thus, there have been significant spatial clusters in that period which means that European countries with a similar number of confirmed cases (high-high or low-low) have been nearer to each other than those with dissimilar counts. But afterwards, there have not been such significant clusters. The significance of this spatial perspective is crucial in understanding the spatial spread of COVID-19 on a country level, particularly in these instances where neighboring countries exhibit similar counts. Geographical place is a crucial component of disease modeling, and this metric may assist in understanding and planning around spatial homogeneities driven by infected persons and their random- and non-random social interactions (20).

## Discussion

At first glance, the model fitting results look promising. We focus the reader's specific attention to the forecast of *N*(*t*), the absolute cumulative counts of the confirmed cases, for different regions in Figures 5, 6. The COVISA19 dashboard provides good modeling for prediction and forecasting. Another important feature is the availability of evaluating the global Moran's index for spatial analysis. Using the provided p-values (e.g., Figure 7, in our paper) of this test, it is possible to survey the spatial correlation of regions under study (15).

There are some other dashboards available for visualization/analyzing the COVID-19 data. Among them, only the ones created by the Johns Hopkins University and the WHO are selected for the comparison purpose. Our reason for this limitation is that the former is believed to be the first dashboard deployed for the public use and the latter is a reference dashboard introduced by the WHO. There are other dashboards available as well, but does not fit meaningfully into the current benchmarked scope as outlined in Table 1 (for example, covid19za-dash (https://bitly.com/covid19za-dash) and https://webs.iiitd.edu.in/raghava/coronavir/).

**Table 1:** Characteristics of some existing web-based dashboards^a^.

**Dashboard Characteristics**	**Johns Hopkins University dashboard**	**WHO's dashboard**	**COVISA19 dashboard**
URL	https://arcg.is/0fHmTX	https://covid19.who.int	https://mahdisalehi.shinyapps.io/Covid19Dashboard
Loading	Slow (**√**)	Fast (**√**)	Relatively fast (**√**)
Comparison over time	**X**	**√**	**√**
Report relative counts	**X**	**X**	**√**
Reports outbreak for each country	**√**	**X**	**√**
Reports outbreak for each continent	**X**	**√**	**√**
Report recoveries	**X**	**X**	**√**
Time series plots	**√** (only for the world)	**√** (only for the top 12 affected countries)	**√**
Moran's I	**X**	**X**	**√**
Dynamic growth models	**X**	**X**	**√**
Map	**√**	**√**	**√**
Dynamic plots	**√**	**√**	**√**
Plots downloadable	**X**	**X**	**√**

a *(√ indicates yes and X indicates no)*.

The value of this dashboard lies in its statistical contribution: the distinction of absolute- vs. relative counts as an important conversation point to assist in distinguishing the severity of COVID-19 per capita of any considered countries within this big data set. For the end-user, this provision assists in maintaining an “apples-to-apples” comparison. The addition of dynamic growth models and Moran's I provides meaningful statistical modeling to gain insight into a data-driven idea of potential future outcomes within the COVID-19 paradigm. Furthermore, this paper illustrated the feasibility of the implementation of R Shiny as a platform for representing big health data (in particular for the case of COVID-19) to any end user. The accessibility of information from this dashboard may be of interest and value not only to healthcare administrators, but also to the general public (as part of general public health awareness) as well as the media who is reporting on the COVID-19 incidence on a global scale see (2, 10, 21, 22). We plan to continue capitalizing on this effective accessibility method using R Shiny to further develop statistical metrics and other inference to further describe, analyses, and understand COVID-19 data.

Github link: https://github.com/Mahdi-Salehi-PhD/COVID-19-dashboard.

Dataset: https://pomber.github.io/covid19/timeseries.json.

Operating system and programming language: Platform independent (web-browser based), R Shiny.

## Data Availability Statement

The datasets presented in this study can be found in online repositories. The names of the repository/repositories and accession number(s) can be found in the article/supplementary material.

## Author Contributions

MS, MA, and AB: conceptualization. MS: data curation. MS, MA, AB, and JF: formal analysis and methodology. MA, AB, and D-GC: funding acquisition. MS, FE, and MF: software. AB and JF: writing—original draft. MS, MA, AB, JF, and D-GC: writing—review and editing.

## Conflict of Interest

The authors declare that the research was conducted in the absence of any commercial or financial relationships that could be construed as a potential conflict of interest.
